# A Herpes Simplex Virus Type 2 Deleted for Glycoprotein D Enables Dendritic Cells to Activate CD4^+^ and CD8^+^ T Cells

**DOI:** 10.3389/fimmu.2017.00904

**Published:** 2017-08-09

**Authors:** Angello R. Retamal-Díaz, Alexis M. Kalergis, Susan M. Bueno, Pablo A. González

**Affiliations:** ^1^Millennium Institute on Immunology and Immunotherapy, Departamento de Genética Molecular y Microbiología, Facultad de Ciencias Biológicas, Pontificia Universidad Católica de Chile, Santiago, Chile; ^2^Departamento de Endocrinología, Escuela de Medicina, Facultad de Medicina, Pontificia Universidad Católica de Chile, Santiago, Chile; ^3^INSERM U1064, Nantes, France

**Keywords:** vaccine, dendritic cells, dendritic cell function, herpes simplex virus type 2, adaptive immunity, attenuation, T cell activation

## Abstract

Herpes simplex virus type 2 (HSV-2) is highly prevalent in the human population producing significant morbidity, mainly because of the generation of genital ulcers and neonatal encephalitis. Additionally, HSV-2 infection significantly increases the susceptibility of the host to acquire HIV and promotes the shedding of the latter in the coinfected. Despite numerous efforts to create a vaccine against HSV-2, no licensed vaccines are currently available. A long-standing strategy, based on few viral glycoproteins combined with adjuvants, recently displayed poor results in a Phase III clinical study fueling exploration on the development of mutant HSV viruses that are attenuated *in vivo* and elicit protective adaptive immune components, such as antiviral antibodies and T cells. Importantly, such specialized antiviral immune components are likely induced and modulated by dendritic cells, professional antigen presenting cells that process viral antigens and present them to T cells. However, HSV interferes with several functions of DCs and ultimately induces their death. Here, we propose that for an attenuated mutant virus to confer protective immunity against HSV *in vivo* based on adaptive immune components, such virus should also be attenuated in dendritic cells to promote a robust and effective antiviral response. We provide a background framework for this idea, considerations, as well as the means to assess this hypothesis. Addressing this hypothesis may provide valuable insights for the development of novel, safe, and effective vaccines against herpes simplex viruses.

## Introduction

Herpes simplex virus type 2 (HSV-2) infects nearly 500 million people worldwide and is the main cause of genital ulcers in symptomatic individuals (1, 2). Importantly, infection may be transferred to neonates during birth, which may lead to life-threatening encephalitis (3). Although antivirals limit HSV-2 replication in the newborn, serious long-term neurologic sequelae may follow, despite treatment (4–6). HSV-2 is persistent in humans, establishing latency in neurons with periodic symptomatic or asymptomatic reactivations that shed infectious virus and significantly contribute to the spread of HSV-2 in the population (7, 8). Importantly, the risk of acquiring HIV is 3-fold higher among individuals that are HSV-2-seropositive (9, 10). In regions where HSV-2 infection is highly prevalent, it is estimated that nearly 50% of HIV infections may be attributed to previous HSV-2 infections (10, 11). Although oral antivirals limit the extent of the HSV reactivations, reduce virus shedding and shorten the duration of herpetic lesions, these drugs do not resolve persistent infection (12, 13). Thus, vaccines that prevent primary infection, block reactivation, and virus shedding are wanted to limit the spread of HSV-2 in the population and its multiple deleterious effects. Although important efforts have been undertaken for developing a vaccine against this virus and HSV-1, regretfully these attempts have failed so far.

## HSV-2 Vaccine Approaches

Subunit vaccine candidates consisting of glycoprotein D from HSV-2 (gD-2), alone or in combination with other HSV envelope glycoproteins, as well as different adjuvants have predominated the HSV vaccine field for nearly 20 years (14–16). Such vaccine development efforts have mainly focused on gD-2 as the main viral target and are likely based on the fact that this glycoprotein is conserved among HSV-2 and HSV-1 isolates (17) and is essential for the entry of the virus into target cells both, immune and non-immune (18, 19). Additionally, HSV-2-infected individuals display high titers of anti-gD-2 antibodies indicating that this viral protein is highly immunogenic and highly visible to the immune system (20, 21). However, although antibodies directed against gD-2 after vaccination or natural exposure to the virus may display neutralizing activity *in vitro* (16, 22–25), their antiviral effects *in vivo* have seemingly been overestimated, as their presence in individuals not necessarily correlates with protective immunity (22, 26–28).

Because subunit vaccine candidates have failed so far at eliciting protective immunity against HSV-2 in clinical trials, other more traditional approaches, such as those based on attenuated mutant viruses have re-emerged as prophylactic alternatives for eliciting immunity against this virus (29). The notion that an attenuated HSV may achieve protective immunity against HSV-2 could be somewhat based on the fact that a weakened herpes virus, namely the varicella zoster virus Oka strain is currently used as a protective and therapeutic vaccine against varicella and shingles (30, 31). Nevertheless, its efficacy is modest, and it may be replaced in the short term by a subunit-based vaccine (32, 33). However, because attenuated HSV mutants have been relatively poorly explored as potential vaccines against HSV-2 this approach should be revisited.

At present, several attenuated viruses have been shown to be safe and confer protective immunity against HSV-2 in animal models. One example is an HSV mutant that has the nuclear localization sequence of the viral protein ICP0 deleted (0ΔNLS), which has been shown to be attenuated *in vivo* and induces protective antibodies targeted against numerous viral proteins (Table 1) (34–36). Another virus exhibiting very positive results *in vivo* and shown to confer protection against HSV infection, is an HSV-2 mutant virus designated dl5-29, which has U_L_5 and U_L_29 deleted (Table 1) (37–39). Both attenuated viruses are being further studied in animal models and have transitioned into clinical trials (see Rational Vaccines Inc. and https://clinicaltrials.gov, respectively). Other attenuated HSV mutants that elicit protective immunity against infection with HSV in animal models are a mutant virus deleted at *U_L_39* which was designated ICP10ΔPK, because it has the protein kinase domain of the large subunit of HSV-2 ribonucleotide reductase (ICP10) deleted (40–43), an HSV-2 virus that has mutations in gD (44) which limit neuron infection, and an HSV mutant that has glycoprotein E (gE) deleted (45). Other mutant viruses tested as potential vaccines in animal models are HSV-1 VC2, which is a glycoprotein K (*U_L_53* gene) and envelope protein U_L_20 (*U_L_20* gene)-deficient virus (46), AD 472 which has *U_L_55-56* (γ_1_*34.5* gene), *U_L_43.5*, and the *U_S_10-12* region deleted (47) and finally RAV 9395, a mutant virus with *U_L_55* and *U_L_56* genes deleted (48). Noteworthy, HSV-1 VC2 was recently tested in macaques with promising results (46).

**Table 1:** HSV-2 mutants tested as attenuated virus vaccines in animal models.

HSV mutant	Deletion or mutation	Outcome	Reference
HSV-2 Δ*gD*^−/+gD1^	Glycoprotein D (*U_S_6* gene) deleted from the genome. Complemented phenotypically with gD-1 (VD60 cells)	Protects against genital and skin challenges and blocks neuronal infection. Confers cross-protection against HSV-1. Antibody-mediated protection	(49, 50)
HSV-2 ΔTK	Viral thymidine kinase (TK, *U_L_23* gene) deleted	Protective, although non-optimal immunity in the mouse genital HSV infection model	(51, 52)
HSV-2 ICP10ΔPK	Protein kinase domain (PK) of the large subunit of HSV-2 ribonucleotide reductase (ICP10) deleted	Induction of a Th1 response and CD8^+^ cytotoxic T lymphocytes	(42)
HSV-2 Δ*gH*^−/+gH^	Glycoprotein H (*U_L_22* gene) deleted from the genome. Complemented phenotypically with gH (Vero F cells)	Protection in the guinea pig model. Tested on individuals with symptomatic HSV-2. Neither virus shedding or recurrences rates were affected	(53, 54)
HSV-2 RAV 9395	*U_L_55, U_L_56*, and *RL1* genes deleted (deletion was done on both copies of the γ_1_34.5 gene *RL1*)	Reduction in herpetic lesions and severity in the guinea pig model. Stimulates both, cell-mediated and humoral immune responses	(48)
HSV-2 dl5-29	DNA replication helicase (*U_L_5* gene) and Infected Cell Protein 8 (ICP8, Major DNA-binding protein, *U_L_29* gene) deleted	Induces neutralizing antibodies and virus-specific CD8^+^-T cell responses in mice. Conferred protection in the guinea pig model	(55, 56)
HSV-2 AD 472	*U_L_55-56, RL1* (gene for γ_1_34.5), *U_L_43.5*, and the *U_S_10-12* region deleted	Humoral and cellular immune response in mice and reduced frequency of herpetic reactivation in the guinea pig model	(47)
HSV-2 ΔgE	Envelope glycoprotein E (*U_S_8* gene) deleted	Reduced vaginal disease, viral titers, neuronal infection. However, protection was incomplete in the mouse infection model	(45)
HSV-2-gD27	Point mutations in the Nectin-1 binding domain of gD-2 (D215G, R222N, and F223I)	Impaired at infecting neurons. Provides protection in the mouse model, despite inducing modest titers of HSV-2-neutralizing antibodies in the serum	(44)
HSV-1 VC2	Glycoprotein K (*U_L_53* gene) and Envelope protein U_L_20 (*U_L_20* gene) deleted	Induced protection through both, humoral and cellular responses in mice and conferred protection against genital challenges with HSV-1 and HSV-2 in rhesus macaques	(46)
HSV-2 0ΔNLS	Nuclear localization signal of the E3 ubiquitin-protein ligase ICP0 protein (*RL2* gene) deleted	Induces protection in the mouse model through antibodies directed against numerous viral proteins. Elicits an antibody response against glycoprotein B and ICP viral proteins	(34, 35, 57)

Importantly, all these mutants have shown to be attenuated in animals (safe) and elicit either HSV-specific antibodies or HSV-specific T cells, or both, and confer protection against HSV-2 infection.

Because gD is essential for virus entry into target cells, deletion of this gene (ΔgD) should likely result in an attenuated virus that is impaired at infecting cells. Yet, if such virus is phenotypically complemented with gD protein on the surface of the virion, it would be capable of infecting cells, although its replication and progeny would likely be hampered. A virus with such characteristics (ΔgD^−/+gD1^) was recently created and tested in animals and shown to be safe, highly immunogenic and confer protection against later challenges with high doses of clinical isolates of HSV-1 and HSV-2 in the skin and genital tissue (49, 50). These somewhat unexpected results may be partially explained by the fact that gD has been previously described to inhibit T cell proliferation and induce their death (58–61). Additionally, gD has been reported to decrease the cytotoxic activity of NK cells (62). Together, these findings suggest that gD may negatively modulate the induction of an effective antiviral response and thus, its deletion from the virion may promote more favorable immune responses.

Noteworthy, a mutant virus with glycoprotein H (*U_L_22* gene) deleted (ΔgH) conferred protection against primary HSV infection and reduced recurrent disease symptoms in an HSV infection animal model (53). However, once tested in a clinical setting it failed to show a therapeutic response (Phase II clinical trial) (54). Another HSV mutant that displays incomplete protection *in vivo* after subcutaneous vaccination is a virus that is deleted for the viral thymidine kinase (TK). Indeed, although this mutant virus elicits anti-HSV antibodies and antiviral T cells when inoculated subcutaneously, these immune components only confer partial protection to genital infection in the mouse infection model (52).

Although most of the attenuated viruses described above elicit significant levels of protection in HSV animal infection models, which are mediated by adaptive immune components, such as anti-HSV antibodies and antiviral T cells, a specific correlate of protection against HSV has not been identified to date. Hence, it is currently unknown what would make a particular vaccine formulation better than another at conferring immunity and which one would have increased chances of being successful once applied to humans.

## Dendritic Cell Infection with HSV-2

Dendritic cells are professional antigen presenting cells that play fundamental roles at establishing and regulating immune responses at the interface of innate and adaptive immunity (63). DCs are distributed within organs and tissues in the body and also located in the periphery, in tissues such as the skin and genital tract where they detect, capture, and process microbes and their antigens (64). Upon antigen capture, these cells migrate to the draining lymph nodes and display peptide fragments obtained from these microorganisms to antigen-specific T cells (63, 65). This DC–T cell interaction will educate T cells based on the integration of signals derived from membrane-bound and soluble molecules presented and secreted by DCs (66, 67). Attributes developed in T cells include the capacity to kill infected cells, secrete modulatory cytokines and regulate the functions of other immune cells, which will ultimately define the overall profile of the immune response elicited against an antigen (68). Therefore, many pathogens have evolved molecular mechanisms to hamper the function of DCs (69–71).

**Figure 1:**
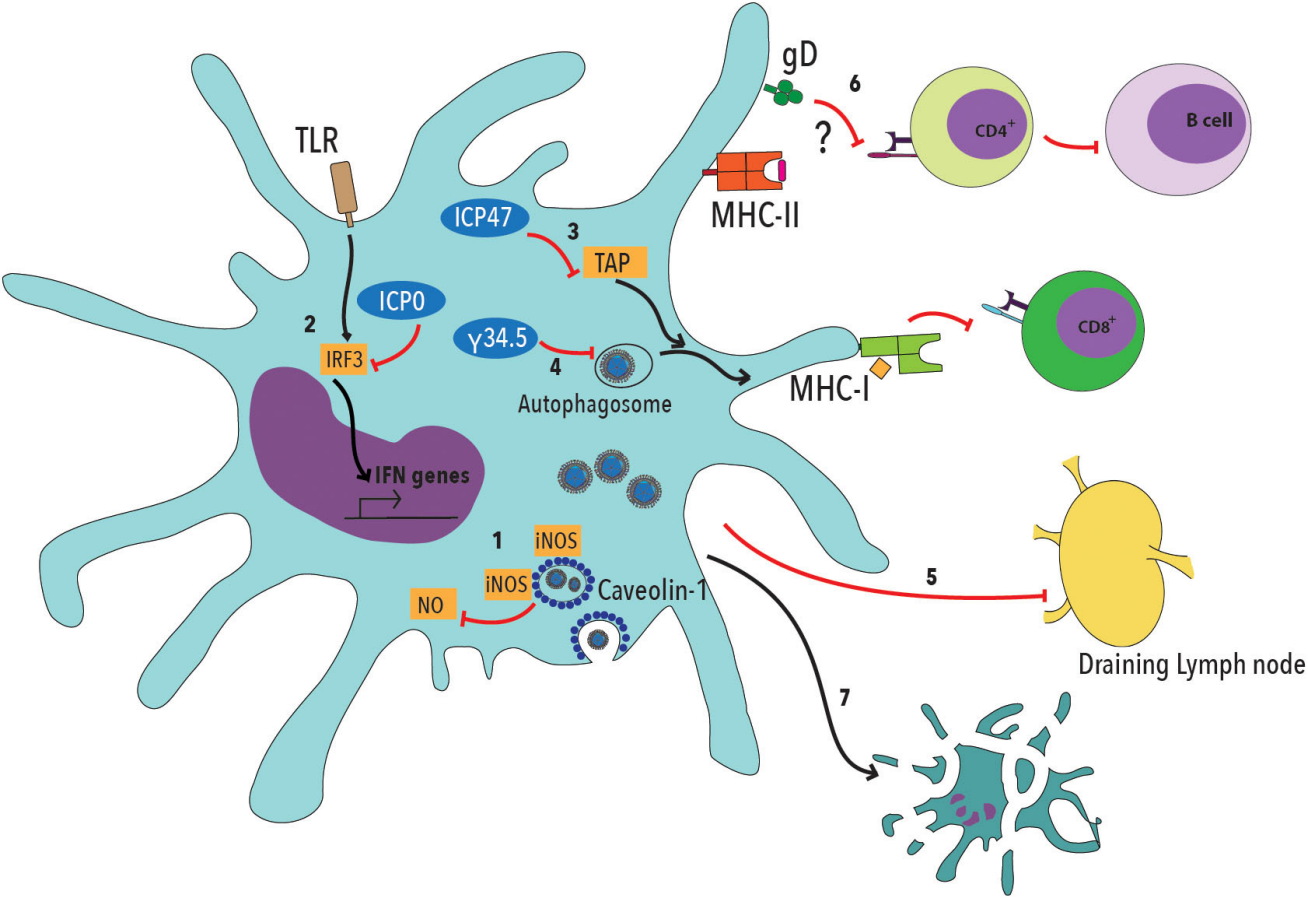
Herpes simplex viruses negatively modulate dendritic cell function. 1. HSV viral proteins bind caveolin-1 and sequester inducible nitric oxide synthase (iNOS), dampening NO production within these cells, which has been described to be involved in cellular antiviral responses. 2. Viral ICP0 interferes with TLR-IRF3 signaling, thus reducing type-I interferon (IFN-I) production by DCs. 3. The HSV ICP47 viral protein interferes with peptide translocation from the cytoplasm to the endoplasmic reticulum, which is mediated by TAP (Transport Associated with Antigen Processing), thus decreasing antigen presentation to CD8^+^ T cells on MHC-I molecules. 4. HSV protein γ34.5 blocks autophagosome maturation, thus reducing the capacity of DCs to process viral antigens. 5. DC infection with HSV interferes with the migration of these cells to the draining lymph nodes, where T cell activation takes place. 6. Glycoprotein D (gD) has been previously described to decrease T cell activation by negatively modulating TCR signaling. Such inhibition likely affects both, CD4^+^ and CD8^+^ T cells. 7. Finally, DC infection with HSV elicits apoptosis.

Noteworthy, HSV can interfere with DC function by blocking their maturation, migration to lymph nodes, promote the secretion of proinflammatory cytokines and inhibit normal autophagosome activity (Figure 1) (72–76). Furthermore, HSV-2 can limit DC presentation of viral antigens on MHC-I molecules by interfering with the transport of antigenic peptides from the cytoplasm to the endoplasmic reticulum and decrease the expression of T cell costimulatory molecules on the DC surface, thus impeding effective T cell activation (Figure 1) (74, 75, 77, 78). HSV can also block the activity of inducible nitric oxide synthase (iNOS) and NO production, by interacting with caveolin-1 (Cav-1) in DCs (Figure 1) (79). Additionally, and most importantly, HSV-2 elicits DC apoptosis early after infection, further limiting the chances of the host to establish an optimal antiviral T cell response (Figure 1) (17, 78, 80).

A recent report assessed the interaction of DCs with an attenuated HSV-1 mutant that induces protective immunity *in vivo*. Importantly, it was found that this virus was attenuated in DCs *in vitro* (i.e., innocuous, non-lethal) (81). Interestingly, the mutant virus expressed viral proteins in these cells despite limited genome replication, which was ultimately abrogated in these cells. Infection with this mutant virus was followed by DC maturation. Recently, we observed similar results with the ΔgD^−/+gD1^ mutant virus (Retamal-Díaz et al., unpublished data). We found that this mutant virus was non-lethal to DCs and expressed non-structural proteins in these cells, despite the fact that viral genome replication was hampered. Furthermore, we found that although the ΔgH virus was attenuated *in vivo*, it was lethal to DCs in *in vitro* assays (Retamal-Díaz et al., unpublished data).

## Statement of Hypothesis

Based on the key role of DCs in establishing effective antiviral responses against pathogenic microbes and the negative effects that HSV-2 exerts over these cells, we hypothesize that: *Attenuated viruses that confer protective immunity in vivo, namely those that rely on adaptive immune components, are attenuated in dendritic cells*. Similarly, the opposite may also be a valid hypothesis: *HSV-2 mutant viruses that are attenuated in DCs confer protective immunity in vivo*. We believe that such hypotheses have not been previously considered, at least not in an explicit manner. A recent study that assess the interaction of an attenuated HSV-1 mutant with DCs *in vitro*, which correlates with its protective properties *in vivo* is in line with this hypothesis (81), as well as data from our laboratory with the ΔgD^−/+gD1^, as described above. Thus, the hypothesis mentioned previously can be more specifically narrowed to: *A herpes simplex virus type 2 deleted for glycoprotein D that is safe in vivo and confers protective immunity, is attenuated in DCs and enables these cells to activate CD4^+^and CD8^+^T cells*, which could also apply to other HSV mutant viruses that confer protection in animal models (Table 1).

## Assumption

For this hypothesis to be consistent with previous reports, we consider that an important assumption should be taken into consideration. This assumption proposes that for the HSV-DC interaction to be considered as proestablishing protective immunity, the mutant virus should be able to express numerous of its genes within these cells, despite potential arrest of viral genome replication. This proposed condition arises from the observation that immunizations with UV-inactivated viruses are not protective, a finding which is true for either wild-type or mutant and attenuated viruses (50, 81). Data recently reported by others and data from our group support this notion (Retamal-Díaz et al., unpublished data) (81). Furthermore, this idea may be supported, at least partially by the fact that the expression of viral gene products from the virus genome will enable DCs to present a wider spectrum of viral antigens to T cells, extending beyond those present in the virion. Indeed, HSV encode at least 70 genes within 150,000 bp DNA, with half of the gene products present in the virion (82). Transcription and translation of viral genes within infected DCs may also translate into increased amounts of viral antigens being loaded by these cells onto MHC molecules for presentation to T cells.

## Suggested Experimental Tests

To assess the abovementioned hypotheses, we suggest a series of experiments with the HSV-2 ΔgD^−/+gD1^ mutant virus, as well as other HSV mutants described in Table 1. All these mutant viruses have shown to confer at least some degree of protection to challenges with virulent HSV *in vivo*, in different HSV infection models. We propose that similar experiments also be performed with HSV mutants that have not shown satisfactory vaccination results *in vivo*. Interestingly, both human and murine DCs succumb to the negative effects of HSV-1 and HSV-2 and thus, either type of DC could be used in these assays, although experimenting with human DCs will likely be considered more insightful. The parental wild-type virus for each one of the mutant viruses assessed should be included in the experiments.

The experiments proposed below should allow assessing a wide range of key functions in DCs that are typically hampered after infections with wild-type virulent HSV.

### DC Viability

DC viability has been previously shown to be severely compromised after inoculation with HSV-1 or HSV-2 (72, 80). This effect is observed at multiplicity of infections (MOIs) as low as 0.1. Hence, DCs may be inoculated with the different HSV viruses outlined in Table 1 and assessed for viability 24 and 48 h later with increasing MOIs by methods such as resazurin (e.g., AlamarBlue^®^) and viability dyes (e.g., Live/Dead^®^, Zombie^®^) that are fixable and can be safely assessed by flow cytometry. It is expected that viruses that have the potential to elicit robust protective immunity *in vivo*, will not significantly affect DC viability.

### DC Maturation

DC maturation is the process by which these cells acquire a phenotype that promotes T cell activation. DC maturation usually relates to the expression of numerous activation markers on the surface of these cells, such as the expression of antigen-presenting molecules MHC-I and MHC-II, and T cell costimulatory molecules such as CD80, CD86, and CD83 (72, 83, 84). Additionally, DC maturation relates to the secretion of soluble molecules, namely cytokines such as IL-6, IL-10, IL-12, and TGF-β, which educate T cells and polarize them. Indeed, cytokine secretion by DCs not only evidences their maturation status, but also informs on the likelihood of the phenotype of T cells resulting from this interaction (67, 85). Other markers of DC maturation include increased antigen degradation, which may be assessed with exogenously added fluorescent-labeled proteins (e.g., labeled-ovalbumin) and the production of reactive oxygen species, which has been related to increased antigen-degradation capacity (86).

### DC-Mediated T Cell Activation

Although anti-HSV T cells can be detected within HSV-infected humans (22, 87–89) and animals (49, 90–92) after natural exposure to the virus or infection with wild-type virus, numerous studies show that HSV hamper the capacity of DCs to activate these cells *in vitro* (72, 74, 80, 93). Furthermore, vaccination studies frequently show that T cell responses in animals vaccinated with protective formulations, such as attenuated HSV mutants, are enhanced as compared to those observed in animals challenged with wild-type virus alone. Hence, *in vitro* T cell activation by DCs may be considered as a readout of the DC–T cell activating capacity *in vivo* and an approach to quantify the magnitude of this activation (67, 94–98). Thus, to assess the potential of DCs pulsed with the mutant viruses to elicit protective T cell responses, virus-pulsed DC–T cell cocultures may be performed. For this, transgenic mouse antigen-specific T cells that either recognize viral antigens or exogenously added antigens can be used. Fortunately, there currently exist transgenic mice that harbor HSV-specific CD8^+^ T cells that recognize a HSV glycoprotein B (gB)-derived peptide on MHC-I (99) and HSV-specific CD4^+^ T cells that recognize a gD-derived peptide on MHC-II (100). Obviously, the latter could not be used for DCs inoculated with the ΔgD virus, as this virus does not encode gD. In this case, other transgenic mice that recognize foreign antigens on MHC-II, such as a peptide derived from the ovalbumin protein [e.g., OT-II (71)] may be used by pulsing DCs with the corresponding peptide at the time of coculture. Common readouts for T cell activation include the measurement of IL-2 secreted by T cells using ELISA, as well as the expression of CD69 and CD25 on the surface of these cells 24–48 h after coculture by flow cytometry (71).

### DC Migration *In Vivo*

Recent studies indicate that HSV likely impairs DC function by interfering with their migration from the site of infection to the draining lymph nodes (101). Overall interference with this process, or the migration of specific DC subsets that are optimal for T cell activation in the lymph nodes seems to be due, at least in part, to the capacity of HSV to induce DC death (102). To assess whether protective HSV mutants recover this important DC function, or even enhance their migration, assays that consist on the local injection of virus and tracking dyes in the footpads or hind flank of limbs may be performed. These assays allow for the quantification of DC subsets that migrate from the periphery into the lymph nodes after infection (103). We expect HSV mutants that elicit protective immunity to promote the migration of DCs from the site of inoculation to the draining lymph nodes, either by increasing the amount of DCs that reach this secondary lymphoid organ or the migration of DC subtypes that are related to enhanced T cell activation (100, 101, 104).

### DC-Mediated T Cell Activation *In Vivo*

A somewhat simple way to determine if an HSV mutant promotes enhanced DC function *in vivo* is to assess their capacity to activate T cells in this context, after interacting with mutant viruses. For this, DCs can be pulsed *ex vivo* with the viruses of interest and then inoculated into the animal to test for T cell activation (81). To exclude the possibility that T cell activation is occurring because inoculated virus-pulsed DCs that are moribund are being captured by non-infected DCs at the site of inoculation, the viability of DCs inoculated with the mutant virus needs to be verified before transferring these cells into the animals (78). If the mutant virus promotes DC function, these adoptively transferred DCs should promote T cell activation *in vivo*. T cells to be assessed in the animal can be those from the endogenous T cell repertoire, but also may be specific to HSV antigens, either using MHC-multimers or HSV-specific T cells, such as those outlined above to follow the small populations of T cells that are known to recognize HSV antigens (99, 100). Again, several T cell markers may be assessed which account for T cell activation, such as surface markers (e.g., CD69, CD25, CD71), intracellular cytokines (e.g., IL-2, interferon-gamma), and T cell proliferation, which may be assessed using a CFSE-dilution assay measured by flow cytometry (105). Furthermore, transferred cells may be followed thanks to endogenous surface markers, such as CD45.1/CD45.2 (52).

### DC-Mediated Anti-HSV Protective Immunity

To assess the capacity of DCs inoculated with mutant HSV viruses to confer protective immunity against later exposure to HSV, we propose performing adoptive transfer experiments that consist on the injection of mutant virus-treated DCs into animals that will then be challenged with virulent HSV. Importantly, this type of assay devises numerous variables that require attention, such as the amount of DCs being transferred into the animals, the route of administration of these cells, and the number of transfers to be performed, among others. Furthermore, DCs from different sources may be used, such as *in vitro* differentiated DCs from the bone marrow (106, 107), DCs isolated from the spleens of naïve mice which then can be inoculated *ex vivo* with the mutant viruses (108, 109) or DCs purified from mice shortly after vaccination (81). Indeed, a recent report describes the use of the latter method for obtaining DCs inoculated with a mutant HSV-1 virus, particularly from the spleens 3 days after vaccination. These DCs were then transferred into naïve mice in three separate injections, which conferred protection (increased animal survival) against a challenge with a lethal dose of HSV-1 administered through the intranasal route (81). Importantly, we consider that for these assays in which virus-inoculated DCs are transferred into animals, an experimental group consisting of animals being transferred with DCs treated with wild-type virulent virus should also be included, as such treatment may confer some degree of protection to the animals because bystander DCs in the recipient animals could capture apoptotic HSV-infected inoculated DCs from the donor and present their antigens to immune cells (78). Noteworthy, the selection of a particular experimental method will depend, among others on the HSV infection model being evaluated (i.e., genital, skin, nasal or ocular infection, among others). Nevertheless, protective immunity after challenge can be measured, for instance by evaluating viral loads in neuronal and non-neuronal tissues (plaque forming units and viral loads by qPCR) and tissue pathology, among others (49, 50).

## Conclusion

Although the deleterious effects of HSV over the functions of DCs were described nearly 15 years ago, with these interactions resulting in DC death, to date only few HSV mutants that could become future HSV vaccines have been tested directly on these cells (72, 80). We believe that because DCs are key determinants at mounting protective antiviral adaptive immune responses, namely anti-HSV T cells and B cells that secrete protective antiviral antibodies, assessing the interaction between HSV vaccine candidates and DCs could provide valuable insight for identifying correlates of protection for this pathogen. If this interaction proves to be important for acquiring protective immunity *in vivo*, assessing this outcome could help design and select for HSV mutants that elicit strong and protective anti-HSV immune responses. However, we cannot rule out the possibility that vaccine mutant viruses that induce apoptosis in DCs may be good vaccine candidates, as dying virus-infected DCs may be captured by bystander non-infected healthy DCs that present viral antigens effectively to T cells (78). Although the notion that pro-apoptotic mutant pathogens may promote protective immunity has been proposed before for other microbes (110–112), this scenario may be unlikely favorable in the context of HSV infection, as this outcome would somewhat resemble what already occurs upon natural infection of DCs with wild-type virus (72, 80). Thus, in such cases for apoptotic DCs derived from interactions with mutant viruses to be protective, they would need to differ in their immune-activating properties as compared to apoptotic DCs originating from infections with virulent virus. Such differences could relate to the secretion of cytokines released by dying cells, the expression of danger signals by apoptotic DCs and the repertoire of viral genes expressed in these cells, among others.

Taken together, we believe that the hypotheses proposed above are original and can be assessed. Furthermore, they could provide important insights on the mechanism of protection of certain HSV mutant viruses. Results from a recent study (81) and from our laboratory (Retamal-Díaz et al., unpublished data) suggest that the hypotheses proposed herein are likely plausible.

## Author Contributions

AR-D, AK, SB, and PG wrote and reviewed the manuscript.

## Conflict of Interest Statement

The authors declare that the research was conducted in the absence of any commercial or financial relationships that could be construed as a potential conflict of interest. The reviewer SM and handling Editor declared their shared affiliation.

## References

[1] Paz-Bailey G Ramaswamy M Hawkes SJ Geretti AM. Herpes simplex virus type 2: epidemiology and management options in developing countries. Sex Transm Infect (2007) 83(1):16–22. 10.1136/sti.2006.020966 17098770 PMC2598582

[2] Smith JS Robinson NJ. Age-specific prevalence of infection with herpes simplex virus types 2 and 1: a global review. J Infect Dis (2002) 186(Suppl 1):S3–28. 10.1086/343739 12353183

[3] Looker KJ Magaret AS May MT Turner KME Vickerman P Gottlieb SL Global and regional estimates of prevalent and incident herpes simplex virus type 1 infections in 2012. PLoS One (2015) 10(10):e0140765. 10.1371/journal.pone.0140765 26510007 PMC4624804

[4] Brown ZA Selke S Zeh J Kopelman J Maslow A Ashley RL The acquisition of herpes simplex virus during pregnancy. N Engl J Med (1997) 337(8):509–15. 10.1056/NEJM199708213370801 9262493

[5] Ward KN Ohrling A Bryant NJ Bowley JS Ross EM Verity CM. Herpes simplex serious neurological disease in young children: incidence and long-term outcome. Arch Dis Child (2012) 97(2):162–5. 10.1136/adc.2010.204677 21685219 PMC3256733

[6] Steiner I Benninger F. Update on herpes virus infections of the nervous system. Curr Neurol Neurosci Rep (2013) 13(12):414. 10.1007/s11910-013-0414-8 24142852

[7] Corey L Synergistic copathogens – HIV-1 and HSV-2. N Engl J Med (2007) 356(8):854–6. 10.1056/NEJMe068302 17314346

[8] Celum C Wald A Lingappa JR Magaret AS Wang RS Mugo N Acyclovir and transmission of HIV-1 from persons infected with HIV-1 and HSV-2. N Engl J Med (2010) 362(5):427–39. 10.1056/NEJMoa0904849 20089951 PMC2838503

[9] Freeman EE Weiss HA Glynn JR Cross PL Whitworth JA Hayes RJ. Herpes simplex virus 2 infection increases HIV acquisition in men and women: systematic review and meta-analysis of longitudinal studies. AIDS (2006) 20(1):73–83. 10.1097/01.aids.0000198081.09337.a7 16327322

[10] Suazo PA Tognarelli EI Kalergis AM Gonzalez PA. Herpes simplex virus 2 infection: molecular association with HIV and novel microbicides to prevent disease. Med Microbiol Immunol (2015) 204(2):161–76. 10.1007/s00430-014-0358-x 25209142 PMC7102243

[11] Wald A Link K. Risk of human immunodeficiency virus infection in herpes simplex virus type 2-seropositive persons: a meta-analysis. J Infect Dis (2002) 185(1):45–52. 10.1086/338231 11756980

[12] Johnston C Saracino M Kuntz S Magaret A Selke S Huang M-L Standard-dose and high-dose daily antiviral therapy for short episodes of genital HSV-2 reactivation: three randomised, open-label, cross-over trials. Lancet (2012) 379(9816):641–7. 10.1016/S0140-6736(11)61750-9 22225814 PMC3420069

[13] Whitley RJ Gnann JW Jr Acyclovir: a decade later. N Engl J Med (1992) 327(11):782–9. 10.1056/NEJM199209103271108 1288525

[14] Shin H Iwasaki A Generating protective immunity against genital herpes. Trends Immunol (2013) 34(10):487–94. 10.1016/j.it.2013.08.001 24012144 PMC3819030

[15] Awasthi S Friedman HM. Status of prophylactic and therapeutic genital herpes vaccines. Curr Opin Virol (2014) 6:6–12. 10.1016/j.coviro.2014.02.006 24631871

[16] Bernstein DI Aoki FY Tyring SK Stanberry LR St-Pierre C Shafran SD Safety and immunogenicity of glycoprotein D-adjuvant genital herpes vaccine. Clin Infect Dis (2005) 40(9):1271–81. 10.1086/429240 15825029

[17] Cheshenko N Trepanier JB Stefanidou M Buckley N Gonzalez P Jacobs W HSV activates Akt to trigger calcium release and promote viral entry: novel candidate target for treatment and suppression. FASEB J (2013) 27(7):2584–99. 10.1096/fj.12-220285 23507869 PMC3688744

[18] Johnson DC Baines JD. Herpesviruses remodel host membranes for virus egress. Nat Rev Microbiol (2011) 9(5):382–94. 10.1038/nrmicro2559 21494278

[19] Salameh S Sheth U Shukla D. Early events in herpes simplex virus lifecycle with implications for an infection of lifetime. Open Virol J (2012) 6:1–6. 10.2174/1874357901206010001 22291864 PMC3267084

[20] Nicola AV Willis SH Naidoo NN Eisenberg RJ Cohen GH. Structure-function analysis of soluble forms of herpes simplex virus glycoprotein D. J Virol (1996) 70(6):3815–22. 8648717 10.1128/jvi.70.6.3815-3822.1996 PMC190258

[21] Cairns TM Huang ZY Whitbeck JC Ponce de Leon M Lou H Wald A Dissection of the antibody response against herpes simplex virus glycoproteins in naturally infected humans. J Virol (2014) 88(21):12612–22. 10.1128/JVI.01930-14 25142599 PMC4248929

[22] Belshe RB Leone PA Bernstein DI Wald A Levin MJ Stapleton JT Efficacy results of a trial of a herpes simplex vaccine. N Engl J Med (2012) 366(1):34–43. 10.1056/NEJMoa1103151 22216840 PMC3287348

[23] Corey L Langenberg AG Ashley R Sekulovich RE Izu AE Douglas JM Jr Recombinant glycoprotein vaccine for the prevention of genital HSV-2 infection: two randomized controlled trials. Chiron HSV Vaccine Study Group. JAMA (1999) 282(4):331–40. 10.1001/jama.282.4.331 10432030

[24] Kohl S Charlebois ED Sigouroudinia M Goldbeck C Hartog K Sekulovich RE Limited antibody-dependent cellular cytotoxicity antibody response induced by a herpes simplex virus type 2 subunit vaccine. J Infect Dis (2000) 181(1):335–9. 10.1086/315208 10608784

[25] Mertz GJ Ashley R Burke RL Benedetti J Critchlow C Jones CC Double-blind, placebo-controlled trial of a herpes simplex virus type 2 glycoprotein vaccine in persons at high risk for genital herpes infection. J Infect Dis (1990) 161(4):653–60. 10.1093/infdis/161.4.653 2181031

[26] Zhu XP Muhammad ZS Wang JG Lin W Guo SK Zhang W. HSV-2 vaccine: current status and insight into factors for developing an efficient vaccine. Viruses (2014) 6(2):371–90. 10.3390/v6020371 24469503 PMC3939461

[27] Retamal-Díaz AR Suazo PA Garrido I Kalergis AM González PA Immune evasion by herpes simplex viruses. Rev Chil Infectol (2015) 32(1):58–70. 10.4067/S0716-10182015000200009 25860047

[28] Cairns TM Huang ZY Gallagher JR Lin Y Lou H Whitbeck JC Patient-specific neutralizing antibody responses to herpes simplex virus are attributed to epitopes on gD, gB, or both and can be type specific. J Virol (2015) 89(18):9213–31. 10.1128/JVI.01213-15 26109729 PMC4542347

[29] Retamal-Díaz AR Tognarelli E Kalergis AM Bueno SM González PA Immune evasion by herpes simplex viruses, herpesviridae. In: Ongrádi J, editor. Herpesviridae. Croatia: InTech (2016). p. 105–46.

[30] Quinlivan M Breuer J. Clinical and molecular aspects of the live attenuated Oka varicella vaccine. Rev Med Virol (2014) 24(4):254–73. 10.1002/rmv.1789 24687808

[31] Quinlivan M Breuer J Schmid DS Molecular studies of the Oka varicella vaccine. Expert Rev Vaccines (2011) 10(9):1321–36. 10.1586/erv.11.93 21919621

[32] Marin M Marti M Kambhampati A Jeram SM Seward JF. Global varicella vaccine effectiveness: a meta-analysis. Pediatrics (2016) 137(3):e20153741. 10.1542/peds.2015-3741 26908671

[33] Cunningham AL. The herpes zoster subunit vaccine. Expert Opin Biol Ther (2016) 16(2):265–71. 10.1517/14712598.2016.1134481 26865048

[34] Halford WP Puschel R Gershburg E Wilber A Gershburg S Rakowski B. A live-attenuated HSV-2 ICP0 virus elicits 10 to 100 times greater protection against genital herpes than a glycoprotein D subunit vaccine. PLoS One (2011) 6(3):e17748. 10.1371/journal.pone.0017748 21412438 PMC3055896

[35] Halford WP Geltz J Messer RJ Hasenkrug KJ. Antibodies are required for complete vaccine-induced protection against herpes simplex virus 2. PLoS One (2015) 10(12):e0145228. 10.1371/journal.pone.0145228 26670699 PMC4682860

[36] Geltz JJ Gershburg E Halford WP. Herpes simplex virus 2 (HSV-2) infected cell proteins are among the most dominant antigens of a live-attenuated HSV-2 vaccine. PLoS One (2015) 10(2):e0116091. 10.1371/journal.pone.0116091 25658852 PMC4319894

[37] Da Costa X Kramer MF Zhu J Brockman MA Knipe DM. Construction, phenotypic analysis, and immunogenicity of a UL5/UL29 double deletion mutant of herpes simplex virus 2. J Virol (2000) 74(17):7963–71. 10.1128/JVI.74.17.7963-7971.2000 10933704 PMC112327

[38] Mundle ST Hernandez H Hamberger J Catalan J Zhou C Stegalkina S High-purity preparation of HSV-2 vaccine candidate ACAM529 is immunogenic and efficacious in vivo. PLoS One (2013) 8(2):e57224. 10.1371/journal.pone.0057224 23468943 PMC3582571

[39] Diaz FM Knipe DM Protection from genital herpes disease, seroconversion and latent infection in a non-lethal murine genital infection model by immunization with an HSV-2 replication-defective mutant virus. Virology (2016) 488:61–7. 10.1016/j.virol.2015.10.033 26609935 PMC4744556

[40] Casanova G Cancela R Alonzo L Benuto R A double-blind study of the efficacy and safety of the ICP10PK vaccine against recurrent genital HSV-2 infections. Cutis (2002). 12403316

[41] Aurelian L Kokuba H Smith CC Vaccine potential of a herpes simplex virus type 2 mutant deleted in the PK domain of the large subunit of ribonucleotide reductase (ICP10). Vaccine (1999) 17(15–16):1951–63. 10.1016/S0264-410X(98)00470-8 10217594

[42] Gyotoku T Ono F Aurelian L. Development of HSV-specific CD4+ Th1 responses and CD8+ cytotoxic T lymphocytes with antiviral activity by vaccination with the HSV-2 mutant ICP10DeltaPK. Vaccine (2002) 20(21–22):2796–807. 10.1016/S0264-410X(02)00199-8 12034107

[43] Wachsman M Kulka M Smith CC Aurelian L. A growth and latency compromised herpes simplex virus type 2 mutant (ICP10DeltaPK) has prophylactic and therapeutic protective activity in guinea pigs. Vaccine (2001) 19(15–16):1879–90. 10.1016/S0264-410X(00)00446-1 11228357

[44] Wang K Goodman KN Li DY Raffeld M Chavez M Cohen JI. A herpes simplex virus 2 (HSV-2) gD mutant impaired for neural tropism is superior to an HSV-2 gD subunit vaccine to protect animals from challenge with HSV-2. J Virol (2015) 90(1):562–74. 10.1128/JVI.01845-15 26559846 PMC4702532

[45] Awasthi S Zumbrun EE Si H Wang F Shaw CE Cai M Live attenuated herpes simplex virus 2 glycoprotein E deletion mutant as a vaccine candidate defective in neuronal spread. J Virol (2012) 86(8):4586–98. 10.1128/JVI.07203-11 22318147 PMC3318599

[46] Stanfield BA Pahar B Chouljenko VN Veazey R Kousoulas KG. Vaccination of rhesus macaques with the live-attenuated HSV-1 vaccine VC2 stimulates the proliferation of mucosal T cells and germinal center responses resulting in sustained production of highly neutralizing antibodies. Vaccine (2017) 35(4):536–43. 10.1016/j.vaccine.2016.12.018 28017425

[47] Prichard MN Kaiwar R Jackman WT Quenelle DC Collins DJ Kern ER Evaluation of AD472, a live attenuated recombinant herpes simplex virus type 2 vaccine in guinea pigs. Vaccine (2005) 23(46–47):5424–31. 10.1016/j.vaccine.2005.02.028 15950327 PMC2718572

[48] Spector FC Kern ER Palmer J Kaiwar R Cha T-A Brown P Evaluation of a live attenuated recombinant virus RAV 9395 as a herpes simplex virus type 2 vaccine in guinea pigs. J Infect Dis (1998) 177(5):1143–54. 10.1086/515278 9592996

[49] Petro C Gonzalez PA Cheshenko N Jandl T Khajoueinejad N Benard A Herpes simplex type 2 virus deleted in glycoprotein D protects against vaginal, skin and neural disease. Elife (2015) 4:e06054. 10.7554/eLife.06054 25756612 PMC4352706

[50] Petro CD Weinrick B Khajoueinejad N Burn C Sellers R Jacobs WR Jr HSV-2 DeltagD elicits FcgammaR-effector antibodies that protect against clinical isolates. JCI Insight (2016) 1(12):e88529 10.1172/jci.insight.88529 27536733 PMC4985247

[51] McDermott MR Smiley JR Leslie P Brais J Rudzroga HE Bienenstock J Immunity in the female genital tract after intravaginal vaccination of mice with an attenuated strain of herpes simplex virus type 2. J Virol (1984) 51(3):747–53. 6088797 10.1128/jvi.51.3.747-753.1984 PMC255840

[52] Shin H Iwasaki A. A vaccine strategy that protects against genital herpes by establishing local memory T cells. Nature (2012) 491(7424):463–7. 10.1038/nature11522 23075848 PMC3499630

[53] Boursnell ME Entwisle C Blakeley D Roberts C Duncan IA Chisholm SE A genetically inactivated herpes simplex virus type 2 (HSV-2) vaccine provides effective protection against primary and recurrent HSV-2 disease. J Infect Dis (1997) 175(1):16–25. 10.1093/infdis/175.1.16 8985191 PMC7109964

[54] de Bruyn G Vargas-Cortez M Warren T Tyring SK Fife KH Lalezari J A randomized controlled trial of a replication defective (gH deletion) herpes simplex virus vaccine for the treatment of recurrent genital herpes among immunocompetent subjects. Vaccine (2006) 24(7):914–20. 10.1016/j.vaccine.2005.08.088 16213066

[55] Da Costa XJ Jones CA Knipe DM. Immunization against genital herpes with a vaccine virus that has defects in productive and latent infection. Proc Natl Acad Sci U S A (1999) 96(12):6994–8. 10.1073/pnas.96.12.6994 10359827 PMC22033

[56] Hoshino Y Dalai SK Wang K Pesnicak L Lau TY Knipe DM Comparative efficacy and immunogenicity of replication-defective, recombinant glycoprotein, and DNA vaccines for herpes simplex virus 2 infections in mice and guinea pigs. J Virol (2005) 79(1):410–8. 10.1128/JVI.79.7.4554.2005 15596834 PMC538700

[57] Halford WP Puschel R Rakowski B. Herpes simplex virus 2 ICP0 mutant viruses are avirulent and immunogenic: implications for a genital herpes vaccine. PLoS One (2010) 5(8):e12251. 10.1371/journal.pone.0012251 20808928 PMC2923193

[58] La S Kim J Kwon BS Kwon B. Herpes simplex virus type 1 glycoprotein D inhibits T-cell proliferation. Mol Cells (2002) 14(3):398–403. 12521303

[59] Yang Y Wu S Wang Y Pan S Lan B Liu Y The Us3 protein of herpes simplex virus 1 inhibits T cell signaling by confining linker for activation of T cells (LAT) activation via TRAF6 protein. J Biol Chem (2015) 290(25):15670–8. 10.1074/jbc.M115.646422 25907557 PMC4505477

[60] Sloan DD Han JY Sandifer TK Stewart M Hinz AJ Yoon M Inhibition of TCR signaling by herpes simplex virus. J Immunol (2006) 176(3):1825–33. 10.4049/jimmunol.176.3.1825 16424213

[61] Vanden Oever MJ Han JY. Caspase 9 is essential for herpes simplex virus type 2-induced apoptosis in T cells. J Virol (2010) 84(6):3116–20. 10.1128/JVI.01726-09 20071584 PMC2826057

[62] Grauwet K Cantoni C Parodi M De Maria A Devriendt B Pende D Modulation of CD112 by the alphaherpesvirus gD protein suppresses DNAM-1-dependent NK cell-mediated lysis of infected cells. Proc Natl Acad Sci U S A (2014) 111(45):16118–23. 10.1073/pnas.1409485111 25352670 PMC4234607

[63] Banchereau J Briere F Caux C Davoust J Lebecque S Liu YJ Immunobiology of dendritic cells. Annu Rev Immunol (2000) 18:767–811. 10.1146/annurev.immunol.18.1.767 10837075

[64] Duluc D Gannevat J Joo H Ni L Upchurch K Boreham M Dendritic cells and vaccine design for sexually-transmitted diseases. Microb Pathog (2013) 58:35–44. 10.1016/j.micpath.2012.11.010 23201532 PMC3596496

[65] Trombetta ES Mellman I. Cell biology of antigen processing in vitro and in vivo. Annu Rev Immunol (2005) 23:975–1028. 10.1146/annurev.immunol.22.012703.104538 15771591

[66] Steinman RM Hemmi H. Dendritic cells: translating innate to adaptive immunity. Curr Top Microbiol Immunol (2006) 311:17–58. 17048704 10.1007/3-540-32636-7_2

[67] Gonzalez PA Carreno LJ Figueroa CA Kalergis AM. Modulation of immunological synapse by membrane-bound and soluble ligands. Cytokine Growth Factor Rev (2007) 18(1–2):19–31. 10.1016/j.cytogfr.2007.01.003 17344089

[68] Mempel TR Henrickson SE Von Andrian UH. T-cell priming by dendritic cells in lymph nodes occurs in three distinct phases. Nature (2004) 427(6970):154–9. 10.1038/nature02238 14712275

[69] Bueno SM Riquelme S Riedel CA Kalergis AM Mechanisms used by virulent *Salmonella* to impair dendritic cell function and evade adaptive immunity. Immunology (2012) 137(1):28–36. 10.1111/j.1365-2567.2012.03614.x 22703384 PMC3449244

[70] Moutaftsi M Mehl AM Borysiewicz LK Tabi Z. Human cytomegalovirus inhibits maturation and impairs function of monocyte-derived dendritic cells. Blood (2002) 99(8):2913–21. 10.1182/blood.V99.8.2913 11929782

[71] Gonzalez PA Prado CE Leiva ED Carreno LJ Bueno SM Riedel CA Respiratory syncytial virus impairs T cell activation by preventing synapse assembly with dendritic cells. Proc Natl Acad Sci U S A (2008) 105(39):14999–5004. 10.1073/pnas.0802555105 18818306 PMC2567482

[72] Stefanidou M Ramos I Mas Casullo V Trepanier JB Rosenbaum S Fernandez-Sesma A Herpes simplex virus 2 (HSV-2) prevents dendritic cell maturation, induces apoptosis, and triggers release of proinflammatory cytokines: potential links to HSV-HIV synergy. J Virol (2013) 87(3):1443–53. 10.1128/JVI.01302-12 23152529 PMC3554174

[73] Raftery MJ Winau F Kaufmann SH Schaible UE Schonrich G. CD1 antigen presentation by human dendritic cells as a target for herpes simplex virus immune evasion. J Immunol (2006) 177(9):6207–14. 10.4049/jimmunol.177.9.6207 17056550

[74] Gobeil PA Leib DA Herpes simplex virus gamma34.5 interferes with autophagosome maturation and antigen presentation in dendritic cells. MBio (2012) 3(5):e267–212. 10.1128/mBio.00267-12 PMC3470650 23073763

[75] Elboim M Grodzovski I Djian E Wolf DG Mandelboim O. HSV-2 specifically down regulates HLA-C expression to render HSV-2-infected DCs susceptible to NK cell killing. PLoS Pathog (2013) 9(3):e1003226. 10.1371/journal.ppat.1003226 23555244 PMC3610627

[76] Puttur FK Fernandez MA White R Roediger B Cunningham AL Weninger W Herpes simplex virus infects skin gamma delta T cells before Langerhans cells and impedes migration of infected Langerhans cells by inducing apoptosis and blocking E-cadherin downregulation. J Immunol (2010) 185(1):477–87. 10.4049/jimmunol.0904106 20519652

[77] Hill A Jugovic P York I Russ G Bennink J Yewdell J Herpes simplex virus turns off the TAP to evade host immunity. Nature (1995) 375(6530):411–5. 10.1038/375411a0 7760935

[78] Bosnjak L Miranda-Saksena M Koelle DM Boadle RA Jones CA Cunningham AL. Herpes simplex virus infection of human dendritic cells induces apoptosis and allows cross-presentation via uninfected dendritic cells. J Immunol (2005) 174(4):2220–7. 10.4049/jimmunol.174.4.2220 15699155

[79] Wu B Geng S Bi Y Liu H Hu Y Li X Herpes simplex virus 1 suppresses the function of lung dendritic cells via caveolin-1. Clin Vaccine Immunol (2015) 22(8):883–95. 10.1128/CVI.00170-15 26018534 PMC4519715

[80] Jones CA Fernandez M Herc K Bosnjak L Miranda-Saksena M Boadle RA Herpes simplex virus type 2 induces rapid cell death and functional impairment of murine dendritic cells in vitro. J Virol (2003) 77(20):11139–49. 10.1128/JVI.77.20.11139-11149.2003 14512561 PMC224953

[81] Ma Y Chen M Jin H Prabhakar BS Valyi-Nagy T He B. An engineered herpesvirus activates dendritic cells and induces protective immunity. Sci Rep (2017) 7:41461. 10.1038/srep41461 28150813 PMC5288694

[82] Dolan A Jamieson FE Cunningham C Barnett BC McGeoch DJ The genome sequence of herpes simplex virus type 2. J Virol (1998) 72(3):2010–21. 9499055 10.1128/jvi.72.3.2010-2021.1998 PMC109494

[83] Mott KR Allen SJ Zandian M Akbari O Hamrah P Maazi H Inclusion of CD80 in HSV targets the recombinant virus to PD-L1 on DCs and allows productive infection and robust immune responses. PLoS One (2014) 9(1):e87617. 10.1371/journal.pone.0087617 24475315 PMC3903765

[84] Reske A Pollara G Krummenacher C Katz DR Chain BM. Glycoprotein-dependent and TLR2-independent innate immune recognition of herpes simplex virus-1 by dendritic cells. J Immunol (2008) 180(11):7525–36. 10.4049/jimmunol.180.11.7525 18490753

[85] Verboogen DR Dingjan I Revelo NH Visser LJ ter Beest M van den Bogaart G. The dendritic cell side of the immunological synapse. Biomol Concepts (2016) 7(1):17–28. 10.1515/bmc-2015-0028 26741354

[86] Sheng KC Pietersz GA Tang CK Ramsland PA Apostolopoulos V. Reactive oxygen species level defines two functionally distinctive stages of inflammatory dendritic cell development from mouse bone marrow. J Immunol (2010) 184(6):2863–72. 10.4049/jimmunol.0903458 20176741

[87] Wyckoff JH III Osmand AP Eisenberg RJ Cohen GH Rouse BT. Functional T cell recognition of synthetic peptides corresponding to continuous antibody epitopes of herpes simplex virus type 1 glycoprotein D. Immunobiology (1988) 177(2):134–48. 10.1016/S0171-2985(88)80034-2 2456985

[88] Long D Skoberne M Gierahn TM Larson S Price JA Clemens V Identification of novel virus-specific antigens by CD4(+) and CD8(+) T cells from asymptomatic HSV-2 seropositive and seronegative donors. Virology (2014) 46(4–465):296–311. 10.1016/j.virol.2014.07.018 25108380

[89] Khan AA Srivastava R Chentoufi AA Geertsema R Thai NT Dasgupta G Therapeutic immunization with a mixture of herpes simplex virus 1 glycoprotein D-derived “asymptomatic” human CD8+ T-cell epitopes decreases spontaneous ocular shedding in latently infected HLA transgenic rabbits: association with low frequency of local PD-1+ TIM-3+ CD8+ exhausted T cells. J Virol (2015) 89(13):6619–32. 10.1128/JVI.00788-15 25878105 PMC4468472

[90] Muller WJ Dong L Vilalta A Byrd B Wilhelm KM McClurkan CL Herpes simplex virus type 2 tegument proteins contain subdominant T-cell epitopes detectable in BALB/c mice after DNA immunization and infection. J Gen Virol (2009) 90(Pt 5):1153–63. 10.1099/vir.0.008771-0 19264627 PMC2675279

[91] St Leger AJ Peters B Sidney J Sette A Hendricks RL. Defining the herpes simplex virus-specific CD8+ T cell repertoire in C57BL/6 mice. J Immunol (2011) 186(7):3927–33. 10.4049/jimmunol.1003735 21357536 PMC3308013

[92] Platt RJ Khodai T Townend TJ Bright HH Cockle P Perez-Tosar L CD8+ T lymphocyte epitopes from the herpes simplex virus type 2 ICP27, VP22 and VP13/14 proteins to facilitate vaccine design and characterization. Cells (2013) 2(1):19–42. 10.3390/cells2010019 24709642 PMC3972665

[93] Peretti S Shaw A Blanchard J Bohm R Morrow G Lifson JD Immunomodulatory effects of HSV-2 infection on immature macaque dendritic cells modify innate and adaptive responses. Blood (2005) 106(4):1305–13. 10.1182/blood-2004-12-4899 15845898 PMC1895187

[94] Rogers PR Song J Gramaglia I Killeen N Croft M. OX40 promotes Bcl-xL and Bcl-2 expression and is essential for long-term survival of CD4 T cells. Immunity (2001) 15(3):445–55. 10.1016/S1074-7613(01)00191-1 11567634

[95] Weatherill AR Maxwell JR Takahashi C Weinberg AD Vella AT. OX40 ligation enhances cell cycle turnover of Ag-activated CD4 T cells in vivo. Cell Immunol (2001) 209(1):63–75. 10.1006/cimm.2001.1783 11414737

[96] Holdorf AD Kanagawa O Shaw AS. CD28 and T cell co-stimulation. Rev Immunogenet (2000) 2(2):175–84. 11258416

[97] Borthwick NJ Lowdell M Salmon M Akbar AN. Loss of CD28 expression on CD8(+) T cells is induced by IL-2 receptor gamma chain signalling cytokines and type I IFN, and increases susceptibility to activation-induced apoptosis. Int Immunol (2000) 12(7):1005–13. 10.1093/intimm/12.7.1005 10882412

[98] Habib-Agahi M Phan TT Searle PF. Co-stimulation with 4-1BB ligand allows extended T-cell proliferation, synergizes with CD80/CD86 and can reactivate anergic T cells. Int Immunol (2007) 19(12):1383–94. 10.1093/intimm/dxm106 17977894

[99] Mueller SN Heath WR McLain JD Carbone FR Jones CM. Characterization of two TCR transgenic mouse lines specific for herpes simplex virus. Immunol Cell Biol (2002) 80(2):156–63. 10.1046/j.1440-1711.2002.01071.x 11940116

[100] Bedoui S Whitney PG Waithman J Eidsmo L Wakim L Caminschi I Cross-presentation of viral and self antigens by skin-derived CD103+ dendritic cells. Nat Immunol (2009) 10(5):488–95. 10.1038/ni.1724 19349986

[101] Kim M Truong NR James V Bosnjak L Sandgren KJ Harman AN Relay of herpes simplex virus between Langerhans cells and dermal dendritic cells in human skin. PLoS Pathog (2015) 11(4):e1004812. 10.1371/journal.ppat.1004812 25875649 PMC4395118

[102] Zhao X Deak E Soderberg K Linehan M Spezzano D Zhu J Vaginal submucosal dendritic cells, but not Langerhans cells, induce protective Th1 responses to herpes simplex virus-2. J Exp Med (2003) 197(2):153–62. 10.1084/jem.20021109 12538655 PMC2193810

[103] Bollampalli VP Nylén S Rothfuchs AG A CFSE-based assay to study the migration of murine skin dendritic cells into draining lymph nodes during infection with *Mycobacterium bovis* bacille Calmette-Guérin. J Vis Exp (2016) (116). 10.3791/54620 PMC5092184 27768071

[104] Hor JL Whitney PG Zaid A Brooks AG Heath WR Mueller SN. Spatiotemporally distinct interactions with dendritic cell subsets facilitates CD4+ and CD8+ T cell activation to localized viral infection. Immunity (2015) 43(3):554–65. 10.1016/j.immuni.2015.07.020 26297566

[105] Belz GT Wilson NS Smith CM Mount AM Carbone FR Heath WR. Bone marrow-derived cells expand memory CD8+ T cells in response to viral infections of the lung and skin. Eur J Immunol (2006) 36(2):327–35. 10.1002/eji.200535432 16402408

[106] Lutz MB Kukutsch N Ogilvie AL Rossner S Koch F Romani N An advanced culture method for generating large quantities of highly pure dendritic cells from mouse bone marrow. J Immunol Methods (1999) 223(1):77–92. 10.1016/S0022-1759(98)00204-X 10037236

[107] Inaba K Inaba M Romani N Aya H Deguchi M Ikehara S Generation of large numbers of dendritic cells from mouse bone marrow cultures supplemented with granulocyte/macrophage colony-stimulating factor. J Exp Med (1992) 176(6):1693–702. 10.1084/jem.176.6.1693 1460426 PMC2119469

[108] Schlecht G Mouries J Poitrasson-Riviere M Leclerc C Dadaglio G. Purification of splenic dendritic cells induces maturation and capacity to stimulate Th1 response in vivo. Int Immunol (2006) 18(3):445–52. 10.1093/intimm/dxh384 16415098

[109] de Heusch M Oldenhove G Urbain J Thielemans K Maliszewski C Leo O Depending on their maturation state, splenic dendritic cells induce the differentiation of CD4(+) T lymphocytes into memory and/or effector cells in vivo. Eur J Immunol (2004) 34(7):1861–9. 10.1002/eji.200424878 15214034

[110] Lau A Singh V Soualhine H Hmama Z. Expression of Cathepsin S in BCG converts it into a pro-apoptotic and highly immunogenic strain. Vaccine (2017) 35(16):2060–8. 10.1016/j.vaccine.2017.02.065 28318770

[111] Li G Liu G Song N Kong C Huang Q Su H A novel recombinant BCG-expressing pro-apoptotic protein BAX enhances Th1 protective immune responses in mice. Mol Immunol (2015) 66(2):346–56. 10.1016/j.molimm.2015.04.003 25942359

[112] Gengenbacher M Nieuwenhuizen N Vogelzang A Liu H Kaiser P Schuerer S Deletion of nuoG from the vaccine candidate *Mycobacterium bovis* BCG DeltaureC:hly improves protection against tuberculosis. MBio (2016) 7(3):e679–616. 10.1128/mBio.00679-16 PMC4895111 27222470

